# Cardiac Interoceptive Accuracy: An Empirical Comparison of Three Ability Measures

**DOI:** 10.1111/psyp.70078

**Published:** 2025-06-04

**Authors:** Eszter Ferentzi, Luca Vig, János Körmendi, Michael Witthöft, Alexander L. Gerlach, Anna Pohl

**Affiliations:** ^1^ Institute of Health Promotion and Sport Sciences ELTE Eötvös Loránd University Budapest Hungary; ^2^ Ádám György Psychophysiology Research Group Budapest Hungary; ^3^ Doctoral School of Psychology ELTE Eötvös Loránd University Budapest Hungary; ^4^ Faculty of Psychology Ruhr University Bochum Bochum Germany; ^5^ Institute of Clinical Psychology and Psychotherapy University of Cologne Cologne Germany

**Keywords:** cardiac interoceptive accuracy, cvSDT, heartbeat counting task, heartbeat discrimination task, heartbeat perception, interoception

## Abstract

There are several measures used to assess one's ability to perceive their heartbeat (cardiac interoceptive accuracy). These can be categorized into two main task types: tracking (e.g., motor tracking, heartbeat counting) and discrimination (e.g., two‐ and multi‐interval). The recently developed cardiovascular signal detection task (cvSDT) combines the advantages of heartbeat counting and multi‐interval discrimination tasks. It is an open question of how the three tasks relate to each other. This study compares all three methods in a sample of young adults (*n* = 73, 66% female). Efforts were made to identify heartbeat perceivers. Expectation and confidence ratings about perceived performance and interoception questionnaires were also administered. We found a relation between tracking and cvSDT(ρ = 0.401, *p* < 0.001); the multi‐interval task was unrelated to both other task types (tracking: ρ = −0.103, *p* = 0.398; cvSDT: ρ = −0.103, *p* = 0.398). Multiple linear regression analyses (with the control of resting heart rate, body fat percentage, and sex) confirmed these results. 27.4% of the sample were heartbeat perceivers according to the heartbeat counting task, 28.8% according to the multi‐interval discrimination task, and 12.3% according to the cvSDT. There was only one heartbeat perceiver according to all three tasks. Among questionnaires and tasks, only one connection was revealed: the Body Awareness Questionnaire related to the bias in cvSDT (ρ = −0.283*, *p* < 0.05). In summary, the three tasks likely assess partly different abilities. The investigation of expectation and confidence also supports this assumption. When choosing the method of cardiac interoception, characteristics should be considered to fit the research question.

## Impact Statement

Measures of heartbeat perception (cardiac interoceptive accuracy), namely the heartbeat counting task, the multi‐interval discrimination task, and the recently developed cardiovascular signal detection task measure different abilities. They differ in the definition of good heartbeat perceivers. Differences are also expressed in their relation to self‐reports (questionnaires, expectancy, and confidence). The two tracing tasks (heartbeat counting and the new task), however, might share some similarities.

## 1 Introduction

Interoception, the processing of internal bodily stimuli, is considered important for homeostatic functions, cognitive abilities, emotional experiences, and therefore mental health conditions (Köteles 2021). The original, narrow definition of interoception covered only the sensations originating in the visceral organs (for a review, see: Ceunen et al. 2016). The definition commonly used today is broader and includes all kinds of bodily sensations (Craig 2002). The importance of interoception is supported by several empirical papers that found a connection between interoception and various emotional and cognitive processes, as well as mental health (for reviews see: Critchley and Garfinkel 2017; Khalsa et al. 2018; Tsakiris and Critchley 2016).

From the beginning of interoception research to the present day, the operationalization of conscious perception of internal signals has been a challenge. Interoception can be measured by focusing on various bodily sensations (e.g., cardiac, gastric, breath‐related), among which the most often measured is heartbeat (Garfinkel et al. 2015). Heartbeats are measured quickly and without much technical effort. Moreover, heartbeat is interesting because it varies according to external and internal demands (e.g., in anxiety‐provoking and stressful situations), indicating sympathetic activation (Steptoe and Vögele 1991; Thayer and Lane 2000).

Heartbeat perception tasks are usually categorized broadly into two main procedures, tracking, and discrimination (Carroll 1977; for more information about the different heartbeat perception procedures and their categorization, see Brener and Ring 2016; Körmendi and Ferentzi 2023). During tracking procedures, the task is to focus on one's heartbeat and either report the counted (or estimated) number of heartbeats (Dale and Anderson 1978; Schandry 1981) or tap a key in synchrony with each heartbeat (Brener 1974; McFarland 1975). Among the tracking procedures, the heartbeat counting task (Schandry 1981) has been used most often. In this task, the correspondence of reported and measured heartbeats determines the interoceptive accuracy score. Critics of this task point out that good performance can be achieved by using strategies that are not based on interoceptive abilities, such as knowledge about heart rate and the estimation based on this information (Desmedt et al. 2020; Phillips et al. 1999; Ring and Brener 1996, 2018; Windmann et al. 1999). Because cardiac sensations are mostly vague, one might not be aware of the majority of heartbeats. This opens the way for response strategies relying on non‐interoceptive processes (e.g., guessing heart rate), as well as response biases (Pohl et al. 2021). Response bias in this context refers to the decision strategies participants apply when they perform the tasks. Heartbeat counting scores are generally dominated by underreports, i.e., the estimated number of heartbeats is 30%–40% lower than the actual number (Ring and Brener 1996), and less than 5% of participants overreport (Zamariola et al. 2018). Thus, presumably a more liberal response bias (the tendency to say ‘yes, there is a heartbeat’ in doubt) leads to reporting larger numbers, closer to the number of recorded heartbeats, hence better scores. Consequently, performance scores drastically reduce when participants are instructed to conduct the task conservatively, not to rely on guessing, and to only report heartbeats clearly felt (Desmedt et al. 2018; Ehlers et al. 1995). Unfortunately, instructions are not always reported in the published studies; according to what is available, there are many different versions.

In discrimination tasks, participants must compare an external rhythm (visual, tactile, or auditory stimuli) with their heartbeats and decide whether they are synchronous (Brener and Jones 1974; Whitehead et al. 1977). The most used method of generating an external stimulus is coupling it with the R‐waves and adding different time delays. Discrimination tasks were mainly implemented as two‐forced‐choice tasks with two types of delay, one delay being defined as synchronous, and one as asynchronous (Katkin et al. 1981, 1982; Störmer et al. 1989; Whitehead et al. 1977); heartbeat perceivers can tell them apart. With the application of signal detection theory, two‐forced‐choice discrimination tasks provide the possibility of separating sensitivity from response bias; however, they are hard to perform (Pohl et al. 2021). Because these tasks are not sensitive to the inter‐individual time point variations of heartbeat perception (i.e., people differ in their particular perception of synchrony in terms of the time‐difference between the R wave and their conscious heartbeat perception), multi‐interval discrimination tasks were developed (Brener et al. 1993; Yates et al. 1985). In these multi‐interval tasks, participants are classified as heartbeat perceivers if their synchronous ratings are unevenly distributed across the different delays and if their rating variance is low (Brener et al. 1993; Ring and Brener 2018; Yates et al. 1985). Although discrimination paradigms do not share the shortcomings of tracking tasks, they raise other methodological problems. For example, they were criticized for being too difficult (Bornemann and Singer 2017; Domschke et al. 2010; Pohl et al. 2021) and for the sensitivity of their indices (Wiens and Palmer 2001). Furthermore, performance in discrimination tasks was shown to be dependent on the sensory modality of the external signal (Schulz et al. 2013).

Thus, empirical evidence supports the idea that tracking and discrimination tasks measure different phenomena (Hart et al. 2013; Knoll and Hodapp 1992; Phillips et al. 1999; Ring and Brener 2018; Schulz et al. 2013), although they are probably not completely independent (Schulz et al. 2021). A recent meta‐analysis of heartbeat counting and discrimination tasks using two delays showed that they are only weakly related (Hickman et al. 2020). These authors noted that there is a lack of studies implementing both multi‐interval and heartbeat‐counting tasks. Furthermore, it would be interesting to relate a heartbeat counting performance score, less affected by response strategies, to performance on multi‐interval discrimination tasks.

Using signal detection theory, the heartbeat counting task was recently adapted to disentangle sensitivity from response bias and reduce the impact of estimation. In the Cardiovascular Signal Detection Task (cvSDT), mental tracking interval lengths are defined by a specific number of heartbeats, counted online, by an algorithm. This allows the presentation of a forced choice response format and makes it possible to disentangle cardiac sensitivity from response strategies (i.e., over or underreport of heartbeats, Pohl et al. 2021). In a first study, the heartbeat counting performance score was positively related to both, sensitivity and a more liberal response strategy in the cvSDT. Individuals with low cardiac interoceptive accuracy (in the cvSDT) appeared to benefit more from adopting more liberal response strategies when solving the heartbeat counting task (i.e., showing less underestimation of heart rate). This reduced underestimation might result from increased knowledge about heart rate and/or a stronger inclination to affirm the occurrence of heartbeats. In other words, a quicker tendency to count heartbeats—whether or not informed by accurate knowledge—was related to high performance in the heartbeat counting task (e.g., thinking, ‘My heart should be beating now’; Study 2 of Pohl et al. 2021). Furthermore, the sensitivity index was higher than in the case of previous signal detection paradigms, which implies an adequate level of task difficulty (Pohl et al. 2021).

Beyond behavioral measurements, self‐reports are often used to operationalize interoception. While questionnaires measure trait‐like aspects of the awareness of various bodily processes (Khalsa et al. 2018), confidence ratings related to a heartbeat perception task and self‐reported expectations refer to a state‐like aspect of one's belief about their performance on the task (Garfinkel et al. 2015; Suksasilp and Garfinkel 2022). All these self‐reports are based on beliefs and linked to previous experiences (Suksasilp and Garfinkel 2022). Performance on different heartbeat perception tasks is usually not, or only weakly related to the interoception questionnaires (Ainley and Tsakiris 2013; Ferentzi, Drew, et al. 2018; Garfinkel et al. 2015; Slotta et al. 2021). Regarding confidence ratings, the results are inconclusive; one study found no link with heartbeat counting (Ehlers et al. 1995), while others discovered a positive relationship only in the case of participants with a high performance on the heartbeat counting and the discrimination task (Garfinkel et al. 2015), or only a weak association between confidence rating and heartbeat counting (Horváth et al. 2021). Self‐reported expectations regarding performance measured before the task, on the other hand, are strongly related to heartbeat counting scores (Körmendi et al. 2021).

This study aims to compare the performance on the cvSDT (Pohl et al. 2021) with representatives of both classic paradigms, that is, with the heartbeat counting task (Schandry 1981) and with the multi‐interval discrimination task (Brener et al. 1993; Ring and Brener 2018). We also compared the behavioral measures with self‐reported interoception, i.e., questionnaires, as well as measures of expectation and confidence ratings.

We hypothesized the sensitivity in the cvSDT to be positively related to the performance on both the heartbeat counting and the multi‐interval discrimination tasks. The response bias of the cvSDT was assumed to be negatively related to the heartbeat counting performance (i.e., participants with a more liberal response tendency should have higher heartbeat counting scores) and to not be related to the performance score of the multi‐interval discrimination task. A weak relation between heartbeat counting performance and multi‐interval discrimination performance was assumed. For a more nuanced comparison of all task types, we aimed to compare the individual classification of heartbeat perceivers versus non‐perceivers. Based on previous results (Ring and Brener 2018), we assumed that the proportion of heartbeat perceivers in the heartbeat counting tracking task would be higher than in the multi‐interval test. Ring and Brener (2018) found that 35% of the participants had scores of 0.8 or better in heartbeat counting, while based on the chi‐square test, 27% of participants were classified as heartbeat perceivers in the multi‐interval test. Furthermore, we expected no significant relationship between questionnaires of interoception and the two classical heartbeat perception methods. Regarding the heartbeat counting task, we expected a strong positive association with expectation and a weak positive association with confidence. We had no hypothesis regarding expectation, confidence, and the multi‐interval discrimination task, as to our knowledge, this topic has not been investigated yet, just like the relation of cvSDT and interoception questionnaires.

## 2 Methods

### 2.1 Participants

Seventy‐five students participated for course credit. Participants with self‐reported depression and/or anxiety disorder diagnoses were excluded. Two participants were excluded because of a large number of extrasystoles. The mean age of the remaining 73 participants was 21.75 (SD = 3.157); 65.8% were female. The average resting heart rate was 78.50 (SD = 12.467), with an average body fat percentage of 26.02 (SD = 8.116). The research was approved by the Research Ethics Committee of ELTE University (reference number: 2019/322‐2). Participants signed an informed consent before the start of the measurement.

### 2.2 Procedure

The language of data collection was Hungarian. The measurement had an online and an experimental phase conducted on two consecutive days. The online questionnaires were filled out at home. For the second phase, participants were asked not to use any psychoactive substances before the experiment. In the laboratory, three electrocardiogram (ECG) electrodes were placed (below the left costal arch, over the manubrium, and the xiphoid process). For the baseline heart rate measurement, the instruction was to lean back, relax, and sit still for 60 s. This was followed by three heartbeat‐perception tasks presented in quasi‐random order: the heartbeat counting task (Schandry 1981), the multi‐interval discrimination task (Ring and Brener 2018), and the cvSDT (Pohl et al. 2021).

The ECG signal was recorded by the Varioport system (Becker Meditec, Karlsruhe, Germany). The Uvariotest software (Gerhard Mutz) was used to run the experiment, as well as collect and process the ECG signal (sampling rate at 512 Hz). An algorithm by Vary was used for online R‐wave detection (Vary 1980).

**FIGURE 1:**
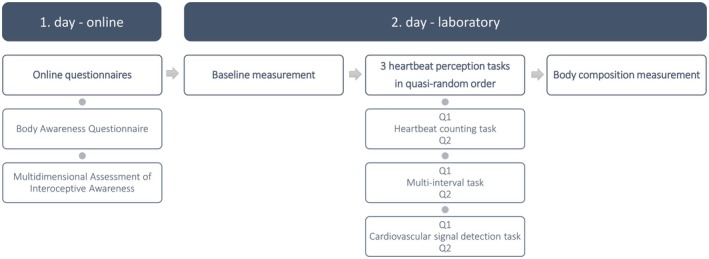
Study protocol. Q1—How accurately do you think you will perceive your heartbeats? Q2—How accurately do you think you perceived your heartbeats?

During the heartbeat‐perception tasks, participants sat upright with both feet on the ground, hands either on their thighs or on the table. All instructions were presented on a screen. The experimenter was present quietly, allowing participants the opportunity to ask questions if the instructions were unclear to them. Expectation and confidence were assessed before and after each task, respectively. Finally, body composition was measured (measuring device: Omron BF511). The study protocol is shown in Figure 1.

### 2.3 Heartbeat‐Perception Tasks

We implemented three heartbeat perception tasks, presented in quasi‐random order. For the exact instructions, see the Supporting Information S1.

### 2.4 Heartbeat Counting Task

During the heartbeat counting task (Schandry 1981), participants had to count their heartbeats silently for different periods (a practice trial, followed by three trials: 25, 35, and 45 s long, presented in random order) without palpating their pulse. Each time interval was marked by a start and an end tone. After each time interval, participants entered the number of counted heartbeats into the computer. No information was provided to the participants regarding the length of the trials or their performance during the experiment.

### 2.5 Multi‐Interval Discrimination Task

The discrimination task applied in this research is a conceptual reproduction of the multi‐interval task by Brener and Ring (Brener et al. 1993; Brener and Ring 2016; Ring and Brener 1992, 2018). During each trial of the task, 10 tones were presented at one of the R‐wave to tone intervals (0, 100, 200, 300, 400, 500 ms). After each trial, participants were required to indicate whether the sounds were synchronous with their perceived heartbeats or not by pressing the corresponding button on a keyboard. The next trial started 5 s after the button press. There were 2 practice trials, followed by 20 of each interval (6 × 20 = 120 trials altogether), presented in a random order (Brener et al. 1993). Because of technical reasons, in this study the 0 ms delay is a delay of 40 ms. It took approximately 40 ms from R‐wave detection to the output of the sound. Because this time delay cannot be corrected online (i.e., during the experiment) for intervals with sound output at R‐wave detection (0 ms), this delay was only corrected for the subsequent intervals (100 ms, 200 ms, etc.).

### 2.6 Cardiovascular Signal Detection Task (cvSDT)

During the cvSDT (Pohl et al. 2021), participants were instructed to count their heartbeats during several time intervals without taking their pulse. After each interval, they had to choose between two options. One was an interval of two beats around the target (e.g., 7–9 in the case of 8 heartbeats), while the other was a response alternative (“less heartbeats” or “more heartbeats”). Either the range (e.g., 7–9) or the response alternative (“less heartbeats”) was correct. There were four practice trials followed by 50 experimental trials. Corresponding to the original protocol, trials with the response alternative “more heartbeats” served as distractors and were not included in the analyses of signal detection parameters. Out of all the experimental trials, only 10 trials included the “more heartbeats” option. In the other 40 trials, each of the two main conditions (“less heartbeats” with the correct range and “less heartbeats” with the incorrect range/correct response alternative) was presented 20 times. Each trial lasted between 7 and 11 heartbeats, which were counted by an online algorithm. Hence there were 5 different trial lengths (equally often represented in each condition). Conditions and interval lengths were presented in a randomized order.

### 2.7 Expectation and Confidence Ratings

Before and after all three heartbeat perception tasks, questions were asked to evaluate their performance. Before it concerned expectancy (“How accurately do you think you will perceive your heartbeats?”), while after, it focused on confidence (“How accurately do you think you perceived your heartbeats?”). Both were assessed using a 10‐point Likert‐type scale.

### 2.8 Questionnaires

#### 2.8.1 Body Awareness Questionnaire

The Body Awareness Questionnaire (BAQ) was developed to measure “normal nonemotive body processes, specifically, sensitivity to body cycles and rhythms, ability to detect small changes in normal functioning, and ability to anticipate bodily reactions” (Shields et al. 1989, 802). The present study used 17 items out of the original 18, based on the recommendation of the Hungarian validation (Köteles 2014). The Cronbach alpha in this study was 0.745.

#### 2.8.2 Multidimensional Assessment of Interoceptive Awareness

Multidimensional Assessment of Interoceptive Awareness (MAIA) was designed to measure various dimensions of interoception, consisting of 8 subscales (Mehling et al. 2012). A recent study argues that it is worth talking about a general MAIA factor that consists of 6 sub‐scales and measures a wider range of interoception (Ferentzi et al. 2020). In this study, the Cronbach alpha of this general factor was 0.80.

### 2.9 Data Management and Statistical Analysis

All ECG data were visually inspected to rule out samples with a large number of extrasystolic events (> 8 extra systoles per minute) and artifacts due to movements or other noise. Due to technical difficulties and movement artifacts, data from some participants were missing for some of the heartbeat perception measurements (for the final sample sizes per measurements see Table 1). Additionally, both in the cvSDT and in the multi‐interval discrimination task, some trials were deleted in the case of three participants each, due to measurement error or movement artifacts. Statistical analysis was conducted using SPSS21 Statistical Software.

**TABLE 1:** Descriptive statistics, normality, and correlation of the heartbeat perception tasks (Spearman's rho).

Variables	*N*	Mean (SD)	Min–Max	*p*‐value of Shapiro–Wilk	HBC rho (*p*)	cvSDT *d*' value rho (*p*)	cvSDT *c* value rho (*p*)
Heartbeat counting task	71	0.70 (0.199)	0.27–0.99	0.004	—	—	—
cvSDT							
*d*' value	70	0.99 (0.585)	0.16–3.13	0.076	0.409 (0.001)	—	—
*c* value	70	−0.18 (0.490)	−1.51—0.85	0.496	0.123 (0.318)	−0.014 (0.905)	—
Multi‐interval discrimination task							
IQR	71	266.96 (29.644)	164.792–321.429	> 0.001	−0.103 (0.398)	−0.183 (0.133)	−0.074 (0.543)

Abbreviations: cvSDT, cardiovascular signal detection task; HBC, heartbeat counting task.

### 2.10 Calculation of Heartbeat Perception Scores

The heartbeat perception score based on the heartbeat counting task was calculated by comparing the number of reported heartbeats with the number of recorded heartbeats and then averaging the score of the three intervals. The following formula was used for each interval: (1 − |(HRrecorded − HRcounted)/HRrecorded|). Higher values indicate better performance. Cronbach's alpha of the 3 trials was α = 0.870.

In the multi‐interval task, heartbeat perceivers and non‐perceivers can be separated by a categorical index. Ring and Brener (Brener et al. 1993; Ring and Brener 2018) applied the chi‐square test to see whether the distribution of synchronous judgment is different from the uniform distribution. It can be assumed that those whose *p*‐value for the chi‐square was more than 0.05 are certainly not detectors (as they judged all the intervals equally often synchronous). Furthermore, heartbeat perception accuracy was calculated by the interquartile range (IQR) of the probability distribution function of the synchronous ratings where the domain (the *X* axis) is the tone interval delays in ms and the codomain (the *Y* axis) is the cumulated number of the presentations considered synchronous (Brener et al. 1993; Ring and Brener 2018). Because this distribution function is discrete, continuous data were generated via linear interpolation (Brener et al. 1993). The temporal location of the heartbeat sensation is indicated by the median of the intervals rated as synchronous (Brener et al. 1993). Brener and Ring also argue that heartbeat perceivers' synchronous ratings follow a *U*‐shaped curve with the lowest synchronous ratings for tones presented directly after the R‐wave and tones presented 500 ms after the R‐wave (Brener et al. 1993; Ring and Brener 2018). Lower IQR values indicate better heartbeat perception.

In the cardiovascular signal detection task, *d*' and *c* values were calculated to apply signal detection theory. Choosing the range, when it correctly represented the measured heartbeats was classified as hit while choosing it in case of the response alternative (“fewer heartbeats”) being correct was classified as a false alarm. From the number of completed trials in each condition and the response choices, hit—and false alarm rates were calculated (e.g., hits/(hits + misses) × 100). We then used the correction of Snodgrass and Corwin (1988) and loglinear transformation (c.f. Appendix 6 of Macmillan and Creelman 2005) to calculate *z*‐scores of hit‐ (H) and false alarm rates (FA). The sensitivity index was calculated by subtracting FAs from Hs (*d*' = [Z(H) − Z(FA)]), the response bias index, by multiplying the sum of Hs and FAs with −0.5 (*c* = −0.5*[Z(hit) + Z(FA)]). Higher *d*' values represent increased sensitivity. A zero *c* score indicates an unbiased response tendency. Negative c values represent a liberal, while positive c values a conservative response bias (Macmillan and Creelman 2005). This approach was an exact replication of the previous protocol (Study 2 of Pohl et al. 2021).

### 2.11 Defining Heartbeat Perceivers and Non‐Perceivers

In the case of the heartbeat counting task, subjects with a heartbeat perception score above 0.85 were considered heartbeat perceivers (Herbert et al. 2010; Pollatos et al. 2005, 2007; Schandry et al. 1993).

In the case of the multi‐interval task, heartbeat perceivers were identified with χ^2^ analysis (*p* < 0.05) (Ring and Brener 2018; Schneider et al. 1998; Wiens and Palmer 2001; Young et al. 2017).

For the cvSDT, we also calculated a continuous variable that shows the proportion of correct replies, using the following formula: (0.5 × (hit rate − false alarm rate) + 0.5). People with a score ≥ 0.85 were regarded as heartbeat perceivers (Macmillan and Creelman 2005).

### 2.12 Statistical Tests for Examining the Association of the Three Tasks

Previous studies calculated correlations (Ring and Brener 2018) or multiple regression analyses to examine the shared variance of different task types (Schulz et al. 2021). Relations of heartbeat perception indices of all three tasks were estimated using correlations. Due to the violation of normality of some variables and correlations, Spearman's rho was calculated. To investigate inter‐task reliability, we calculated Cohen's Kappa (Cohen 1960) for each task pair.

Three regression analyses were conducted to investigate the relationship between the three heartbeat perception tasks. In all three regression analyses, the control variables were resting heart rate, body fat percentage, and sex (1st step). In the 2nd step, performance on the heartbeat counting task was predicted either by IQR of the multi‐interval task or by the *d*, *c* values of the cvSDT and their interaction term in the first and second regression analysis. The interaction term was calculated as the product of the centered value of the *d*' and *c* values. IQR of the multi‐interval task was predicted by *d*' value of the cvSDT in the third regression analysis.

## 3 Results

Table 1 shows the descriptive statistics of the scores of the applied heartbeat perception task, also including the normality test (*p*‐value of the Shapiro–Wilk test) and the correlations between the measured variables. Only the heartbeat counting task and the *d*' value of cvSDT were significantly related (ρ = 0.409, *p* = 0.001).

**FIGURE 2:**
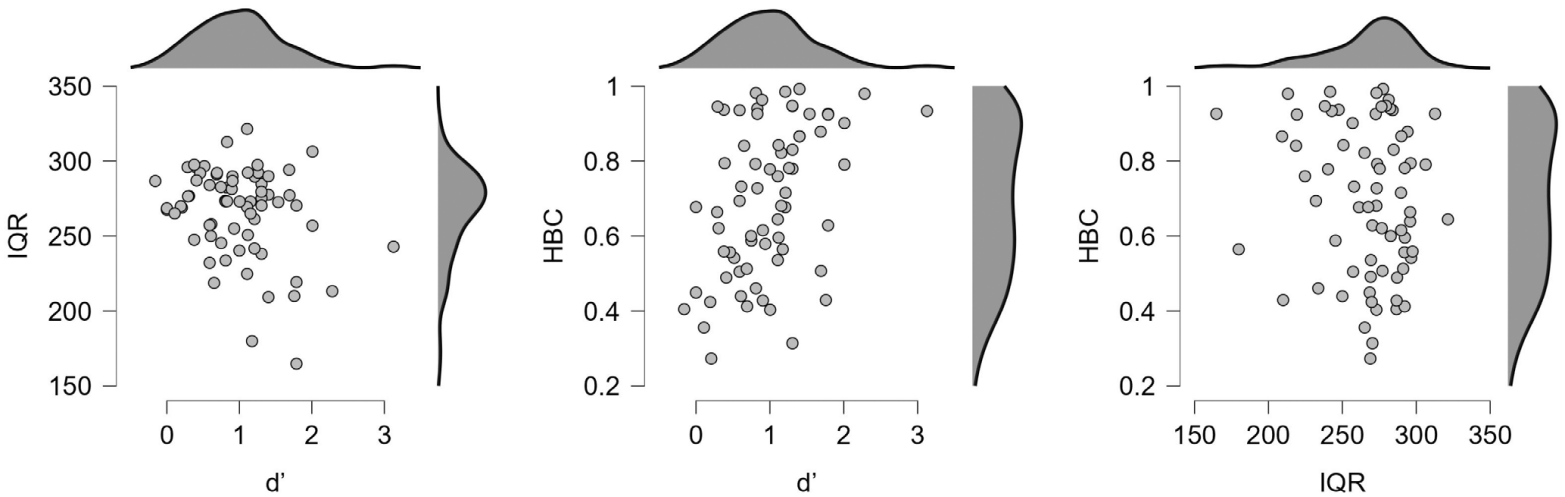
Scatter plots of the variables representing the three heartbeat perception tasks. HBC = score of the heartbeat counting task; *d'* =  d' value of cardiovascular signal detection task; IQR = IQR of the multi‐interval discrimination task.

Figure 2 shows how various heartbeat perception scores relate to each other. Please note that in the case of IQR, a lower value represents better heartbeat perception, while negative *c* values mean liberal; positive *c* values represent conservative response tendencies.

### 3.1 Regressions

In the first regression analysis, the IQR value of the multi‐interval task did not predict the heartbeat counting score, after controlling for resting heart rate, body fat percentage, and sex (see Table 2).

**TABLE 2:** Results of multiple linear regression analysis with IQR as a predictor and the heartbeat counting score as the dependent variable.

	Heart rate	Body fat percentage	Sex	IQR
Step 1 *R* ^2^ = 0.067 *p* = 0.216				
B ± SE	−0.003 ± 0.002	−0.001 ± 0.004	−0.053 ± 0.071	
95.0% CIs	−0.007–0.001	−0.009–0.007	−0.194–0.088	
Standardized β	−0.203	−0.039	−0.127	
*p*	0.098	0.818	0.454	
Step 2 Δ*R* ^2^ = 0.010 *p* = 0.417				
B ± SE	−0.003 ± 0.002	−8.028E‐005 ± 0.004	−0.061 ± 0.071	−0.001 ± 0.001
95.0% CIs	−0.007–0.001	−0.009–0.009	−0.204–0.082	−0.002–0.001
Standardized β	−0.178	−0.003	−0.146	−0.105
*p*	0.160	0.985	0.396	0.417

In the second regression analysis, the *d*' value of the cvSDT task was a significant predictor of the heartbeat counting score, after controlling for resting heart rate, body fat percentage, and sex, and including *c* and *d*' values and their interaction term in the second step. The final equation explained 23.4% of the total variance of the heartbeat counting score (*p* = 0.012; for more details see Table 3).

**TABLE 3:** Results of multiple linear regression analysis with variables of the cvSDT as predictors and the heartbeat counting score as the dependent variable.

	Heart rate	Body fat percentage	Sex	*d'*	*c*	*d'* × *c*
Step 1 *R* ^2^ = 0.065 *p* = 0.239						
B ± SE	−0.003 ± 0.002	−0.001 ± 0.004	−0.047 ± 0.072			
95.0% CIs	−0.007–0.001	−0.010–0.007	−0.192–0.098			
Standardized β	−0.206	−0.046	−0.112			
*p*	0.098	0.791	0.523			
Step 2 Δ*R* ^2^ = 0.169 *p* = 0.008						
B ± SE	−0.001 ± 0.002	−0.001 ± 0.004	−0.033 ± 0.068	0.138 ± 0.043	0.048 ± 0.050	−0.048 ± 0.075
95.0% CIs	−0.005–0.003	−0.009–0.007	−0.168–0.103	0.053–0.224	−0.052–0.148	−0.199–0.102
Standardized β	−0.043	−0.031	−0.078	0.414	0.118	−0.079
*p*	0.729	0.848	0.633	0.002	0.339	0.524

In the third regression analysis, which investigated the relation of multi‐interval task and the cvSDT, the *d*' value did not have a predictive value while body fat percentage had (see Table 4). The final equation explained the 16.0% of the total variance of IQR (*p* = 0.025).

**TABLE 4:** Results of multiple linear regression analysis with d' value of the cvSDT as predictor and the IQR of the multi‐interval task as the dependent variable.

	Heart rate	Body fat percentage	Sex	*d*
Step 1 *R* ^2^ = 0.121 *p* = 0.040				
B ± SE	0.624 ± 0.283	1.256 ± 0.607	−11.985 ± 10.369	
95.0% CIs	0.060–1.189	0.042–2.470	−32.699—8.729	
Standardized β	0.260	0.339	−0.190	
*p*	0.031	0.043	0.252	
Step 2 Δ*R* ^2^ = 0.039 *p* = 0.094				
B ± SE	0.445 ± 0.298	1.218 ± 0.599	−12.996 ± 10.236	−10.730 ± 6.309
95.0% CIs	172.097–291.650	0.020–2.415	−33.451—7.459	−23.338—1.879
Standardized β	0.185	0.329	−0.206	−0.212
*p*	0.140	0.046	0.209	0.094

### 3.2 Heartbeat Perceivers

**FIGURE 3:**
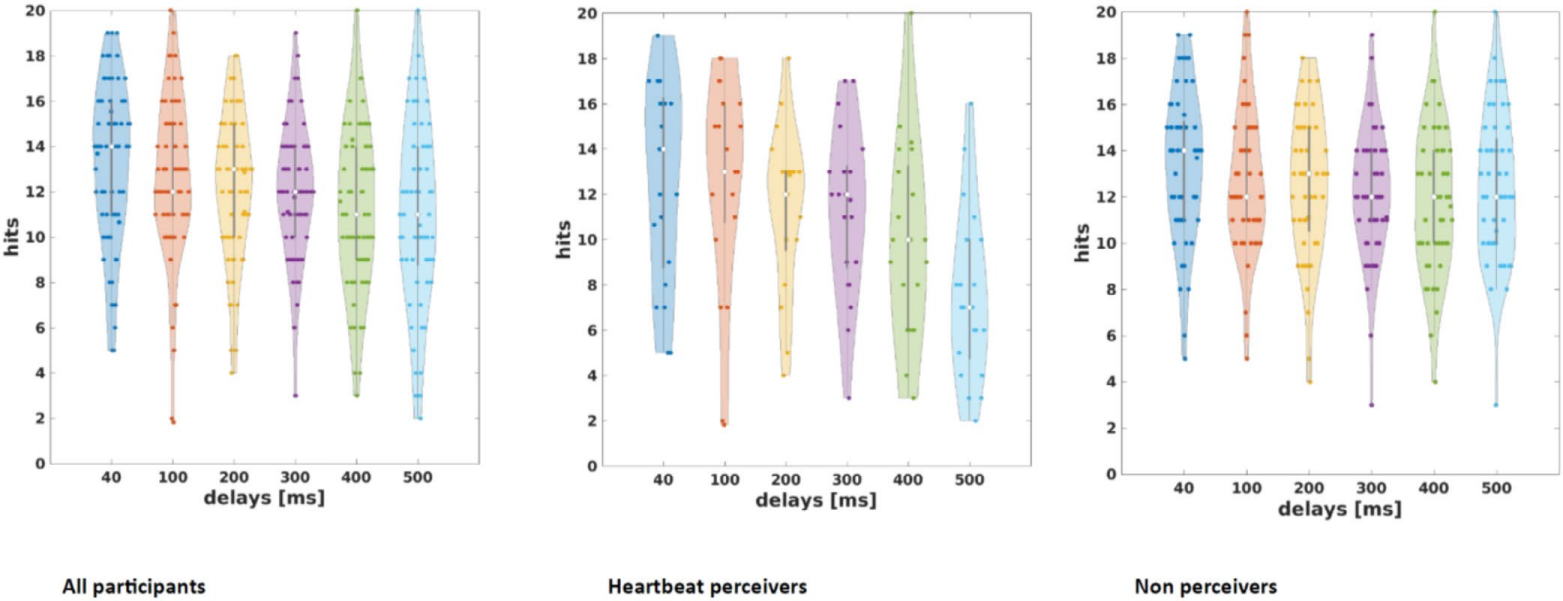
Number of synchronous ratings in the multi‐interval task. Heartbeat perceivers and non‐perceivers were identified with χ^2^ analysis (good: *p* < 0.05).

27.4% (*N* = 20) of the subjects were heartbeat perceivers according to the heartbeat counting task. According to the multi‐interval task, 28.8% of the sample (*n* = 21) were heartbeat perceivers (see Figure 3). In the cvSDT, 12.3% (*n* = 6) were regarded as high performers. There was only 1 person who was considered a heartbeat perceiver according to all three tasks. Cohen's Kappa values were around 0 (heartbeat counting and multi‐interval task: κ = 0.0575; multi interval task and cvSDT: κ = 0.0192; heartbeat counting task and cvSDT: κ = 0.1411), tasks did not show agreement regarding heartbeat perceivers.

In the multi‐interval task, we also applied a new calculation method during which we combined IQR and the chi‐square test. Our logic was that heartbeat perception must be coupled with a low IQR score as well. Thus, we took the average IQR minus one standard deviation as a criterion for heartbeat perception. Among the 21 subjects, 14 (19.2%) had a low IQR as well.

### 3.3 Heartbeat Perception and Self‐Reported Interoception

Table 5 shows the descriptives for the self‐reported interoception variables, i.e., expectation and confidence ratings and questionnaire scores, and their relation to the performance on heartbeat perception tasks.

**TABLE 5:** Self‐reported measures (descriptive statistics, normality) and their correlation with heartbeat perception tasks (Spearman's rho).

	Expectation	Confidence	BAQ	MAIA *g* factor
Mean (SD), min–max			81.53 (10.590) 49–105	3.2 (0.676) 1.101–4.640
Heartbeat counting task	4.75 (1.895), 1–9 ρ = 0.196	4.49 (1.788), 1–8 ρ = 0.420**	ρ = −0.142	ρ = 0.026
cvSDT	4.62 (1.616), 1–8	4.887 (1.809), 1–9		
*d*' value	ρ = 0.137	ρ = 0.227	ρ = 0.104	ρ = 0.121
*c* value	ρ = −0.40	ρ = 0.057	ρ = −0.283*	ρ = −0.178
Discrimination task				
IQR	4.66 (1.549), 1–8 ρ = −0.265*	4.41 (1.869), 1–8 ρ = −0.229	ρ = 0.002	ρ = 0.115

* *p* < 0.05.

** *p* < 0.01.

The negative correlation of expectations regarding the performance on the multi‐interval task with the IQR (ρ = −0.27, *p* = 0.025) indicated a positive association of expectations and task performance. In the case of the heartbeat counting task, confidence rating related moderately to performance (ρ = 0.42, < 0.001). None of the other correlations between heartbeat perception scores and expectation or confidence ratings were significant.

Among questionnaire measures, the g factor of MAIA did not relate to any of the heartbeat perception scores. (For more details about the correlation with all MAIA subscales, see Supporting Information S2). BAQ related to the *c* value of the cvSDT only (ρ = −0.28, *p* = 0.018).

## 4 Discussion

The primary aim of this study was to empirically compare three different heartbeat perception tasks, i.e., the heartbeat counting task (Schandry 1981), the multi‐interval discrimination task (Ring and Brener 2018), and the cardiovascular signal detection task (cvSDT, Pohl et al. 2021). Additionally, we investigated their respective relation to self‐reported interoception (i.e., expectation, confidence, and questionnaire measures). Results indicate that there are some similarities between the two tracking‐based methods (i.e., the heartbeat counting task and the cvSDT). However, findings also demonstrate that the three heartbeat perception tasks are clearly not interchangeable. People with higher scores on the Body Awareness Questionnaire (BAQ, Shields et al. 1989) had lower cvSDT *c* values, i.e., they more probably exhibited a liberal response tendency. People with higher expectations regarding their performance had lower performance scores on the discrimination task, while those with higher confidence after the task had higher performance scores on the heartbeat counting task.

### 4.1 Performance in the Three Heartbeat‐Perception Tasks

Averaged performance indices were in line with previous studies regarding the heartbeat counting task (HBP score: e.g., Pohl et al. 2021; Ring and Brener 2018), the multi‐interval discrimination task (IQR: e.g., Brener et al. 1993; Ring and Brener 2018; Schneider et al. 1998) and the cardiovascular signal detection task (d': Petzke et al. 2024; Pohl et al. 2021). Also, the percentage of detectors according to the chi‐square test was consistent with previous multi‐interval task implementations (Brener et al. 1993; Ring and Brener 2018; Schneider et al. 1998; Young et al. 2017) and similarly high in the heartbeat counting task. According to the cvSDT, the percentage of heartbeat perceivers was considerably lower.

However, the percentage of synchronous ratings did not follow an inverted *U*‐shaped pattern, even among perceivers, as indicated by the chi‐square test. In contrast to previous multi‐interval task implementations, there was no percentage increase from 0 to 200 ms delay (Brener et al. 1993; Ring and Brener 2018; Wiens and Palmer 2001). Similar to previous studies, synchronous ratings dropped at a 500 ms delay (Brener et al. 1993; Ring and Brener 2018; Wiens and Palmer 2001). Wiens and Palmer (2001) plotted the averaged percentages of synchronous judgments of heartbeat perceivers and non‐perceivers and found the 100 ms, 200 ms, and 500 ms best discriminated the two groups. In our study, curves between groups differed only for the 500 ms delay with a lower percentage of synchronous ratings in heartbeat perceivers.

If a reversed *U*‐curve is indicative of proficient heartbeat perception, one could argue that the chi‐square test may be classifying too many individuals as adept perceivers. The fact that distributions that theoretically should not correspond to true heartbeat perception also deviate from the equal distribution has already led other scientists to critically evaluate the chi‐square test (Wiens and Palmer 2001). Our data speak in favor of the reasoning of Wiens and Palmer (2001). Brener and Ring (2016) argue, however, for the possibility of individuals sensing their heartbeat in different body locations in different trials, which may result in multimodal distributions. If this interpretation is applied, the evaluation of the IQRs becomes more difficult. After all, multimodal distributions can also lead to high IQRs, which in turn means that a short IQR is not a reliable parameter for heartbeat perception and therefore the relationship of this parameter with other accuracy indices need not be so pronounced.

### 4.2 Associations Between the Three Heartbeat‐Perception Tasks

As expected, there was a moderate positive relation between the sensitivity value of cvSDT and the performance on the heartbeat counting task, in line with previous results (Pohl et al. 2021). This suggests that the two tasks measure similar cardiac interoceptive ability. The negative relationship between response bias and the heartbeat counting score (Pohl et al. 2021), however, was not replicated. There are several possible explanations for this. Firstly, there is a numerical difference between the mean c value in our sample and the sample in the previous studies (current sample: *c* = −0.18 (SD = 0.2); *c* = 0.23 (SD = 0.45) in Study 2 assessment 1 in Pohl et al. 2021; and *c* = 0.23 (SD = 0.47) in Petzke et al. 2024), which might indicate a more liberal response tendency in the current study. In contrast to the Pohl et al. study, participants worked on three different heartbeat perception tasks in succession. Possibly, the completion of particularly the long multi‐interval task could have affected the performance in the other tasks, in the sense of specific response strategies and impacting the performance of participants in the signal detection and the multi‐interval task. Moreover, the coupling of tones to r‐waves in the multi‐interval task might have increased knowledge about heart rate and led to a reduction of heartbeat underestimation, resulting in a more liberal response strategy in the cvSDT. However, a larger sample would have been needed to conclusively assess sequence or transfer effects.

Regarding the association between cvSDT and the multi‐interval task, performance on the latter was independent of both sensitivity (unexpectedly) and bias (expectedly). Interestingly, participants with higher body fat had higher IQR, i.e., worse performance on the multi‐interval task. This finding is in line with the findings of a recent meta‐analysis concluding that lower interoception is associated with higher BMI (Robinson et al. 2021). Task performance, however, was not influenced by body fat in the case of the two other tasks. It is important to note that BMI and body fat percentage are related but not interchangeable (Deurenberg et al. 1998). Although BMI is more frequently used in interoception studies (Robinson et al. 2021), body fat estimation using bioelectrical impedance is probably a more informative variable because it is likely that the amount of fat affects the perception of bodily signals.

Similarly to body fat, higher heart rates were positively associated with IQR (i.e., lower task performance). The average heart rate of the current sample was slightly higher compared to previous studies, which might have contributed to discrepancies in findings (Ring and Brener 2018; Young et al. 2017).

In line with some previous studies (Hickman et al. 2020; Ring and Brener 2018), but contrary to our hypothesis, the heartbeat counting score was not significantly related to the performance on the multi‐interval task. While during tracking tasks individual heartbeats that do not reach awareness might be ignored following a strict instruction, during discrimination tasks an intuitive decision is made on the validity of a series of stimuli. The sensitivity to non‐conscious sensations is an important difference between tracking (with strict instructions) and discrimination methods; mental tracking tasks catch conscious stimuli only, while tasks applying forced choice can measure the sensation of near‐threshold stimuli.

Previously, authors have argued that the lack of task correlation speaks against the validity of the heartbeat counting task (Brener and Ring 2016; Ring and Brener 2018). This argument is valid only if the multi‐interval task is considered a valid measure of heartbeat perception. However, it is still unclear whether the discrimination tasks differentiate better between heartbeat perceivers and non‐perceivers, compared to the HBP cut‐off. For example, the indices used for assessment are sometimes contradictory (e.g., heartbeat perception in the chi square test does not always go hand in hand with a shorter IQR or an inverted *U*‐curve). Furthermore, it is unclear whether heartbeat perception is a categorical or dimensional construct. If heartbeat perception is categorical, research has to establish reasonable cut‐offs for each heartbeat perception task and compare their classifications. Interestingly, a newly developed method (Plans et al. 2021) called the Phase Adjustment Task works with the assumption that heartbeat perception ability is categorical and classifies people as interoceptive, not interoceptive, or not classifiable.

When choosing a cardiac interoception measure, it should be carefully considered which method fits the research question better. On the one hand, the inclusion of the heartbeat counting task allows for a comparison of results with previous studies; on the other hand, the applied instructions are often not documented, which makes the comparison harder (Ferentzi et al. 2022). One can argue that forced choice tasks (like the cvSDT and the multi‐interval task) might be able to grasp sensations on the border of consciousness. Thus, these methods ought to be chosen if such perceptual processes are important. Additionally, the application of signal detection theory might be useful if response bias is an important factor to take into account, like in the case of symptom perception. Researchers might also consider whether sensitivity to detect perceivers is more important or specificity. While the mental tracking task might be the most sensitive task (classifies perceivers as perceivers but misclassifies none‐perceivers as perceivers), the two‐forced choice discrimination task is probably more specific (misclassifies perceivers as non‐perceivers). Using the multi‐interval task would, in our view, require further examination of the validity of the applied indices (e.g., IQR, chi square test), as well as the clarification of how to differentiate between perceivers and non‐perceivers. Most importantly, as tasks measuring cardiac ability are not interchangeable, one should be careful what conclusions are drawn. It might also be advisable to involve more than one measure of cardiac interoceptive accuracy.

### 4.3 Heartbeat‐Perception Task‐Related Expectations and Confidence

A previous study found that expectation predicts performance in the heartbeat counting task (Körmendi et al. 2021), which was not replicated in our study. Comparably, no relation of expectations and task performance was found in the cvSDT. In our study, the lack of association both in the case of the heartbeat counting task and the cvSDT supports that these two tasks share similar mechanisms. Participants with higher expectations, however, performed better on the multi‐interval task. As far as we know, no previous study related expectations to task performance in the multi‐interval task. When interpreting their own results regarding the heartbeat counting task, Körmendi et al. (2021) emphasize the importance of top‐down processes. Further studies are needed on the role of expectations in the case of heartbeat perception tasks to investigate these (contradictory) findings.

Heartbeat counting scores had a strong link to confidence ratings. An explanation for the link between the heartbeat counting score and the related confidence ratings is that the knowledge about one's heart rate may give a good base for estimation of performance after the task (Horváth et al. 2021). The fact that this guessing technique cannot be applied in the case of the two other applied heartbeat perception tasks might be the reason for the lack of association.

### 4.4 Heartbeat‐Perception Tasks and Questionnaires

No association was expected between questionnaires and any of the heartbeat perception indicators. We found, however, that BAQ was related to response bias in the cvSDT. Interoception questionnaires measure participants' beliefs regarding their ability to perceive their bodies accurately. These beliefs can be seen as perceptual priors in the predictive coding framework that lead to biased perception of bodily processes (Suksasilp and Garfinkel 2022). Our result suggests that this bias corresponds to the bias measured by the cvSDT task. People with liberal response tendencies more readily confirm the perception of a physical signal and describe a bodily feeling as a symptom. Items of BAQ are about the perception of concrete bodily signals; moreover, some of them indicate symptoms or negative bodily sensations (e.g., item 5: “I know in advance when I'm getting the flu.”, Shields et al. 1989). MAIA items, however, grasp a broader interoception concept (Ferentzi et al. 2020; Mehling et al. 2012).

### 4.5 Limitations

One of the limitations of the presented project is that we could not investigate the order effect of the heartbeat perception tasks due to the large number of variations relative to the sample size. It is possible that the order of the tasks influenced the results. We hope, however, that randomization handled this problem partially. Another limitation is that the first version of the MAIA was applied, although a more recent version is now available (Mehling et al. 2018); the Hungarian version of this, however, is not yet available. Furthermore, a possible limitation of the perceiver classification in the cvSDT is that a false alarm rate below 0.15 is easier to achieve by guessing (as compared to the requested hit rate), because false alarms correspond to an overestimation of the number of actual heartbeats. As the cvSDT is a newly developed method, a clear understanding of the task quality requires further investigation.

### 4.6 Implications for Future Research

For future studies, cvSDT might require further adaptations, for example, the reintroduction of a “more‐alternative” reply‐option, where false alarms would correspond to an underestimation of the heart rate. The influence of response strategies on accuracy scores in different heartbeat perception tasks needs further clarification (Desmedt et al. 2023). Future studies could also implement a time estimation task, query participants' knowledge on their own heart rate, and add surveys to explore chosen strategies during task completion qualitatively and quantitatively. Moreover, cvSDT parameters could be investigated in relation to other newly developed heartbeat perception techniques (for reviews see: Desmedt et al. 2023; Körmendi and Ferentzi 2023).

Assessing the heartbeat perception indices under the simulation of perfect heartbeat perception could also provide evidence for task validity. People sensing their heartbeat well in one specific body area should score higher than 85% correct both in the cvSDT and heartbeat counting task, pass the chi‐square test, and produce an inverted *U*‐curve with a low IQR in the multi‐interval task. With the help of those simulations, future research could establish appropriate cut‐offs for the tasks and determine heartbeat perceivers.

Across studies using the multi‐interval task, it would be interesting to analyze how different response strategies might have impacted the distribution of the scores. This analysis would involve the examination of the ratio of synchronous to asynchronous ratings. Individuals inclined toward more conservative responses (fewer synchronicity affirmations) would perhaps yield a reversed *U*‐shaped distribution. In contrast, those with more liberal tendencies (who frequently rate synchronicity) might rather produce more uniform distributions. To the best of our knowledge, the impact of response strategies has not been considered yet.

Taxonometric analyses of heartbeat perception indices can serve as validation strategies (for comparable taxonometric approaches for psychopathological constructs, see Bräscher et al. 2023). The results of these analyses will inform the choice of analyses when heartbeat perception is related to other constructs.

Future studies could also investigate the influence of response strategies during cvSDT. Evidence for liberally biased response strategies in somatic symptom disorders (Wolters et al. 2022), as well as the assumed transdiagnostic relevance of response biases (Van den Bergh et al. 2021) suggests that they might have clinical relevance.

Studies involving more than one modality indicate that interoceptive abilities of different bodily domains may not be related (Crucianelli et al. 2022; Ferentzi, Bogdány, et al. 2018; Ferentzi et al. 2017). The involvement of multiple measures might provide a more complex picture of one's interoceptive ability. Actually, this method has already been applied in a subfield of interoception, when sensory threshold and pain sensitivity are measured with multiple modalities applying Quantitative Sensory Testing (Arendt‐Nielsen and Yarnitsky 2009; Chong and Cros 2004).

## 5 Conclusion

Overall, it is likely that the three investigated tasks require different abilities. The investigation of high performers and self‐reported measures also supports this assumption. Besides, the constructs measured by the two tracking‐based heartbeat perception tasks are probably somewhat overlapping. When choosing a cardiac interoception measure, the pros and cons should be considered to fit the research question.

## Author Contributions

**Eszter Ferentzi:** conceptualization, formal analysis, funding acquisition, methodology, supervision, visualization, writing – original draft, writing – review and editing. **Luca Vig:** conceptualization, data curation, investigation, project administration, writing – original draft, writing – review and editing. **János Körmendi:** data curation, formal analysis, methodology, visualization, writing – review and editing. **Michael Witthöft:** conceptualization, methodology, resources, supervision, writing – review and editing. **Alexander L. Gerlach:** conceptualization, methodology, software, supervision, writing – review and editing. **Anna Pohl:** conceptualization, formal analysis, methodology, software, supervision, validation, writing – original draft, writing – review and editing.

## Disclosure

*Declaration of Generative AI and AI‐Assisted Technologies in the Writing Process*: The authors did not use generative AI technologies for the preparation of this work.

## Conflicts of Interest

The authors declare no conflicts of interest.

## Supporting information


Data S1.

Data S2.

## Data Availability

The data that support the findings of this study are openly available in OSF at https://osf.io/k5twv/.
