# Supplementary material for: Cardiac Interoceptive Accuracy: An Empirical Comparison of Three Ability Measures
Source: Psychophysiology. 2025 Jun 4;62(6):e70078. doi: 10.1111/psyp.70078 (PMC12138236; doi:10.1111/psyp.70078)
Supplement: Supplementary file 1 — Data S1. [file PSYP-62-e70078-s001.docx]

# Supplementary material 1 Instruction of the heartbeat perception tasks

**Instruction of the Heartbeat counting task**

First instruction:

“The following task examines how accurately you can perceive your heartbeats. We will ask you to count your heartbeats in three time intervals without palpating your pulse. Please take off your watch as well. The beginning and the end of each time interval is marked by a tone. After each time interval, your task will be to enter the number of heartbeats you have perceived using the keyboard. This always will be followed by a short pause. Now, you will have a practice trial then the task begins.”

After the first instruction:

“How accurately do you think you will perceive your heartbeats?” Before each interval:

“Now sit back comfortably and as soon you can hear the tone, start counting all the heartbeats you can perceive.”

After each interval:

“Please indicate the number of heartbeats you have perceived. After, press ENTER.” After the practice trial:

“Is everything clear about the task? If so, you can start the experiment by pressing ENTER. If you still have questions, ask the experimenter.”

After the last probe:

“How accurately do you think you perceived your heartbeats?” At the end of the task:

“You have finished this task. Please press ENTER to quit.”

# Instruction of the Multi-interval task

First instruction:

“The following task examines how accurately you can perceive your heartbeats. You will hear tones that indicate heartbeats. Sometimes, these match your heartbeats, other times they are delayed. Please decide after each trial, whether the tones were in synchronous with your heartbeat or not. Now, you will have a practice trial then the task begins. When you are ready, press ENTER.”

After pressing ENTER:

“How accurately do you think you will perceive your heartbeats?” After the subjective accuracy rating:

“Now you will have a practice trial. Please decide after each trial whether the tones were synchronous with your heartbeat or not. When you are ready, press ENTER.”

Before each trial:

“Pay attention to the tones.” After each trial:

“Were the tones synchronous with your heartbeats?” After each decision:

“The next interval will begin shortly.” After the last practice trial:

“The practice trials are over. Please get ready for the experiment phase. If you are ready, press ENTER.”

After the last probe:

“How accurately do you think you perceived your heartbeats?” At the end of the task:

“You have finished this task. Please press ENTER to quit.”

# Instruction of the New cardiovascular signal detection task (cvSDT)

First instruction:

“The following task examines how accurately you can perceive your heartbeats. We ask you to count your heartbeats in different time intervals without palpating your pulse. Please take off your watch as well. The beginning and the end of each time interval is marked by a tone. After each interval, you will see two options on the screen, you can choose between these with the keys 1 and 2. Always choose the option that you think best fits the heartbeats you counted. This will always be followed by a short pause. If you still have questions, ask the experimenter. Press ENTER to continue.”

After pressing ENTER:

“How accurately do you think you will perceive your heartbeats?” After the subjective accuracy rating:

“Now you will have four practice trials. Please, focus to your heartbeat.” Before each trial:

“Lean back comfortably and count all heart beats that you are able to sense after you hear the starting tone.”

After the end tone:

“Please chose between the two options with response button 1 or 2. After, press ENTER” After the last practice trial:

“The practice trials are over. The experiment will begin shortly. Please, focus again to your heartbeats.”

Before the experimental trials:

“Let’s begin. If you do not have any questions, you can start the experiment by pressing ENTER.”

After the last probe:

“How accurately do you think you perceived your heartbeats?” At the end of the task:

“You have finished this task. Please press ENTER to quit.”
