# Supplementary material for: Cardiac Interoceptive Accuracy: An Empirical Comparison of Three Ability Measures
Source: Psychophysiology. 2025 Jun 4;62(6):e70078. doi: 10.1111/psyp.70078 (PMC12138236; doi:10.1111/psyp.70078)
Supplement: Supplementary file 2 — Data S2. [file PSYP-62-e70078-s002.docx]

Supplementary material 2

Correlation coefficients (Spearman’s rho) between MAIA subscales and heartbeat perception tasks (p >.05 in all cases)

| **MAIA**  **subscales** | **Heartbeat perception task** | **cvSDT** | | **IQR** |
| --- | --- | --- | --- | --- |
|  |  | **d’ value** | **c value** |  |
| **Noticing** | .095 | .077 | -.118 | .162 |
| **Not distracting** | -.169 | -.135 | -.064 | .089 |
| **Not worrying** | .050 | .109 | -.116 | .089 |
| **Attention**  **regulation** | .016 | .019 | -.203 | .126 |
| **Emotional**  **awareness** | -.087 | .025 | -.137 | -.032 |
| **Self-**  **regulation** | .063 | .008 | -.057 | .049 |
| **Body listening** | .047 | .074 | -.184 | .004 |
| **Trusting** | .127 | .231 | -.021 | .158 |
